# Medication reconciliation role and value in Alzheimer's disease treatment

**DOI:** 10.1590/0004-282X-ANP-2021-0147

**Published:** 2022-02-28

**Authors:** Xueqian Shen, Qun Zhang, Wenliang Shao, Jianying Shi, Bing Liu

**Affiliations:** 1 The Second Hospital of Jinhua, Department of Pharmacy, Zhejiang Province, China. The Second Hospital of Jinhua Department of Pharmacy Zhejiang Province China; 2 The Second Hospital of Jinhua, Department of Five Wards of Geriatrics, Zhejiang Province, China. The Second Hospital of Jinhua Department of Five Wards of Geriatrics Zhejiang Province China

**Keywords:** Medication Reconciliation, Alzheimer Disease, Safety, Drug-Related Side Effects and Adverse Reactions, Reconciliação Medicamentosa, Doença de Alzheimer, Segurança, Efeitos Colaterais e Reações Adversas Relacionadas a Medicamentos

## Abstract

**Background:**

With the continuous increase of Alzheimer's disease (AD), it is also imminent to treat patients with AD for medication reconciliation.

**Objective:**

To explore the role and value of medication reconciliation in AD treatment.

**Methods:**

100 patients over 65 years of age diagnosed with AD were randomly separated into two groups: conventional treatment and medication reforming. The list of medical orders of all subjects was obtained within 24 hours after admission with Beers criteria, STOPP/START criteria, and Chinese Pharmacopoeia used as the MED intervention criteria. Medication reconciliation was performed at 2 weeks, 1 month, and 2 months after hospital admission. The number of medications prescribed, the quantity of the medication, medication error rate, therapeutic effect, adverse drug reactions, and satisfaction levels of family members and main caregivers were compared between the two groups.

**Results:**

After the intervention, the types and amount of medication in the MED group were less compared to the CON group along with a reduced medication deviation rate. The Mini-mental state examination (MMSE) score and the proportion of well-nourished patients in the MED group were higher than those in the CON group. It was also observed that the physical self-care ability score and the proportion of patients with abnormal swallowing were lower when in comparison with the CON group. The incidence of adverse drug reactions in the MED group was lower than that in the CON group. However, the satisfaction rate was higher than that in the CON group.

**Conclusion:**

Medication reconciliation can reduce the medication deviation in AD patients.

## Resumo

**Antecedentes:**

Com o contínuo aumento da prevalência da doença de Alzheimer (DA), a reconciliação medicamentosa no tratamento de pacientes portadores da doença é também iminente.

**Objetivo:**

Explorar o papel e a importância da reconciliação medicamentosa no tratamento de DA.

**Métodos:**

100 pacientes com mais de 65 anos de idade com diagnóstico de DA foram divididos aleatoriamente em dois grupos: tratamento convencional (CON) e mudança medicamentosa (MED). A relação das prescrições médicas de todos os indivíduos foi obtida dentro das 24 horas após a admissão, usando-se os critérios Beers, os critérios STOPP / START, e a Farmacopéia Chinesa foi usada ​​como intervenção no grupo MED. A reconciliação medicamentosa foi realizada em 2 semanas, 1 mês e 2 meses após a admissão hospitalar. O número de medicamentos prescritos, a quantidade de medicamentos, a taxa de erro de medicação, o efeito terapêutico, as reações adversas a medicamentos e os níveis de satisfação dos familiares e cuidadores principais foram comparados entre os dois grupos.

**Resultados:**

Após a intervenção, os tipos e a quantidade de medicação no grupo MED foram menores em comparação com o grupo CON, juntamente com uma taxa de erro de medicação reduzida. A pontuação do mini-exame do estado mental (MEEM) e a proporção de pacientes bem nutridos no grupo MED foram maiores do que no grupo CON. Observou-se também que o escore da habilidade física de autocuidado e a proporção de pacientes com deglutição alterada foram menores quando comparados ao grupo CON. A incidência de reações adversas a medicamentos no grupo MED foi menor do que no grupo CON. Por outro lado, o índice de satisfação foi superior ao do grupo CON.

**Conclusão:**

A reconciliação medicamentosa pode reduzir os erros de medicação em pacientes com DA.

## INTRODUCTION

With the increasing aging of the population in China, the prevalence of Alzheimer's disease (AD) is also increasing^1^. The majority of patients with Alzheimer's disease have other underlying diseases such as impaired physiological, liver and kidney functions. Compared with other age groups, the absorption, distribution, metabolism and excretion of drugs in the elderly have different characteristics during therapeutic intervention, which can easily lead to drug accumulation and adverse reactions^2^^,^^3^. This could pose a threat to the health and lives of patients. At the same time, taking a large number of drugs could result in poor compliance and expensive treatment, placing a heavy economic burden to families and society and resulting in a waste of medical resources. This has become a highly urgent and vital problem that needs to be solved in the research field of rational drug use. In 2019, the Pharmacy Service Specification for Healthcare Organizations proposed a definition for medication reconciliation: a standardized process of obtaining a complete and accurate list of medications currently being administered to each patient and comparing the medication list with current medical advice to achieve maximum patient care safety^4^.

In 2007, the World Health Organization (WHO), the Joint Commission of the United States (JC), and the Joint Commission International (JCI) jointly issued the "Patient Safety Solutions" to recommend medication reconciliation services for medical institutions. This was done so that patients could receive reasonable drug treatment, reduce medication deviations, and prevent adverse drug reactions^5^. However, the understanding and attention of domestic clinical medical staff, clinical pharmacists, patients, and technologies of drug reorganization did not form a standardized or a normalized service model. Therefore, in this study, through drug reorganization for elderly patients with AD, relevant evaluation tools were used to find possible medication deviations, promote standardized and normalized drug treatment and application, ensure the continuity, rationality, accuracy and safety of patient treatment, save medical resources, reduce economic pressure on patients and their families, and promote long-term benefits.

## METHODS

### Study subjects

AD patients over 65 years of age that were admitted to our hospital from January to June 2020 were selected as the study subjects. The inclusion criteria were: (1) meet AD diagnostic criteria^6^; (2) aged > 65 years and length of hospital stay > 2 months; (3) multiple medications, using more than 5 types of drugs; and (4) patients and their families had knowledge regarding the content of the study and signed the informed consent. 

Exclusion criteria were: (1) patients with severe organic disease; (2) hospice patients; (3) poor compliance, unable to cooperate with allocated treatment; (4) interviewee was not evaluated during the whole process; and (5) unable to obtain the list of previous medication within 48 hours of admission. 

This study was reviewed and approved by the medical ethics committee of the Second Hospital of Jinhua (2019-2-03).

### Medication reconciliation intervention

A medication reconciliation record form was developed with the Beers criteria, STOPP/START criteria^7^ and Chinese Pharmacopoeia^8^ applied as the medication reforming intervention. This was done to perform medication reconciliation and develop a reconciliation plan for elderly patients with AD.

*Collection of medication information*

A comprehensive evaluation form was used to assess patient disease conditions within 24 hours after admission. Clinicians obtained the list of previous therapeutic drugs and the list of therapeutic drugs in the current doctor's advice by checking the patients' self-prepared drugs and by interviewing the patients, their families and main caregivers. Information on the drug name, dose, method of use, adverse drug reactions present during medication and the results of relevant examinations and tests after admission were obtained.

*Analysis of medication information*

Differences in previous drug treatment and medication were compared to current hospitalization according to disease guidelines. Potential inappropriate medications was evaluated and drug reforming was implemented 2 weeks after admission, 1 month after admission, or 2 months after admission according to the treatment needs of patients. The medication reforming program was then given back to the patients, their families, and main caregivers.

### Follow-up

All patients were followed-up before and after medication reforming intervention to understand and record the types, degrees, occurrence time, duration, treatment outcome, course of medication deviations, and adverse reactions. Cognitive function, physical condition (physical function, visual impairment, swallowing function, and muscle quality), nutritional status, and occurrence of adverse drug reactions before and after treatment were also observed and evaluated.

### Evaluation criteria

*Cognitive function evaluation*

The Mini-Mental State Examination (MMSE)^9^ revised was used to evaluate the cognitive function of study subjects before and after medication reforming. This tool includes assessment of orientation, memory and attention, recall ability, and language ability for a calculated total of 30 points.

*Evaluation of physical ability*

The physical ability of the study subjects was evaluated before and after medication reforming using the Activity of Daily Living Scale (ADL)^10^. This scale includes the Physical Self-Care Ability Scale and the Instrumental Activities of Daily Living Scale, using a 4-level scoring method of 1 to 4 points. The higher the score, the higher the degree of decline of function.

*Swallowing function evaluation*

The swallowing function of the study subjects was evaluated before and after medication reforming using the Water swallow test^11^. The patient was placed in a sitting position, and the presence of dysphagia was evaluated by observing and recording the patient's drinking time, drinking status, and the presence or absence of choking cough. The evaluation criteria were classified as grade I to V, with grade I being normal swallowing function, grade II being possible dysphagia, and grade III and V being abnormal swallowing function.

*Nutritional risk assessment*

The evaluation was performed according to the NRS-2002 criteria for nutritional risk screening^12^, including the degree of impact of the disease on the nutritional status of patients, weight changes in the past 3 months, changes in dietary intake in the past 1 week, and Body Mass Index (BMI). The total score of NRS-2002 is 7 points. If the total score is ≥ 3 points, it is considered that the patient has nutritional risk. NRS-2002 < 3 points indicates that the patient has no nutritional risk.

*Safety evaluation*

Routine blood, liver, and kidney function, electrolytes and ECG were measured in all patients before enrolment into the study to evaluate vital signs. The above items were detected again after each medication reforming cycle. The Treatment Emergent Symptom Scale (TESS)^13^ was used for evaluation. If the maximum score of each entry before and after reforming was ≥ 2, it was considered as an "adverse drug reaction". Incidence (%) = number of cases/total number of cases × 100%.

*Satisfaction evaluation*

A self-report satisfaction questionnaire was used to investigate the satisfaction of patients' families and caregivers with drug treatment and reorganization services. The items are rated as "very satisfied", "satisfied", "fair", and "dissatisfied." Overall satisfaction (%) = (very satisfied + satisfied)/total number of cases × 100 %.

### Statistical processing

SPSS 22.0 software was used for statistical analysis. Continuous variables are expressed as mean ± standard deviation (SD). Paired t-test was used for comparison between before and after the intervention. Enumeration data are expressed as n (%) and compared with the ?^*2*^ test. Clinical efficacy grading data were analyzed by rank-sum test. P < 0.05 was considered statistically significant.

## RESULTS

### Patient profile

A total of 100 subjects aged 65 to 89 years (mean one of 82.60 ± 8.47) were enrolled and randomly divided into conventional treatment group (CON, n = 50) and medication reforming group (MED, n = 50) by random number method. Before the intervention, there was no significant difference in age, male sex, BMI, and disease duration between the two groups (Table 1, P > 0.05).

**Table 1.** Comparison of basic data between the two groups.

Group	Age (years)	Male (%)	BMI (kg/m^2^)	Disease course (years)
CON group (n=50)	81.97±8.54	35 (70.0)	23.41±1.25	4.85±1.12
MED group (n=50)	82.76±8.39	32 (64.0)	22.98±1.46	4.56±1.25
t/x^2^	0.470	0.407	1.582	1.222
*P*	0.642	0.523	0.117	0.225

### Medication types and amounts 

After the intervention, the type and amount of medication in the MED group were lower than those in the CON group (P < 0.05). Within MED group, the type and amount of medication after intervention were lower than those before intervention (P < 0.05) (Table 2).

**Table 2.** Comparison of medication types and amounts between the two groups.

Group	Type of medication		Drug price	
	Pre-intervention	Post-Intervention	Pre-intervention	Post-Intervention
CON group (n=50)	6.40±1.85		64.82±10.36	
MED group (n=50)	6.34±1.79	4.20±1.23^#&^	63.99±9.87	43.33±8.42^#&^

Values are reported as mean ± SD. ^#^P<0.05 after intervention; ^&^P<0.05 before and after intervention.

### Potential medication deviations 

A total of 45 potential medication deviations occurred in the 100 patients, with an incidence of 45.0 %. It mainly involved alprazolam, clopidogrel, aspirin and furosemide, and the combination of statins and clopidogrel being the main drug discomfort. There were 32 cases (64.0 %) of potential deviant medication in the CON group and 13 cases (26.0 %) of potential deviant medication in the MED group. There was a significant difference in the potential medication deviation rate between the two groups (P < 0.05), although it was lower in the MED group (Table 3).

**Table 3.** Comparison of potential medication deviation between the two groups.

Group	Missed dose	Incorrect dosage and or administration	Incorrect dosing interval	Additional administration	Repeated administration	Treatment course error	Inappropriate combination of medication	Total potentially deviating medications
CON group (n=50)	15 (30.0)	2 (4.0)	3 (6.0)	7 (14.0)	4 (8.0)	0 (0.0)	1 (2.0)	32 (64.0)
MED group (n=50)	6 (12.0)	0 (0.0)	1 (2.0)	4 (8.0)	2 (4.0)	0 (0.0)	0 (0.0)	13 (26.0)
x^2^								14.586
P								<0.001

Values are reported as n (%).

### Treatment effects between the two groups

*MMSE scores before and after medication*

There was no significant difference in MMSE scores before intervention between the two groups (P > 0.05). After the intervention, the MMSE score in the MED group was higher than that in the intervention group (P < 0.01) (Table 4).

**Table 4.** Comparison of MMSE scores and ADL scores before and after medication reforming between the two groups.

Group	MMSE scores		Physical self-care ability		Instrumental daily living ability	
	Pre-intervention	Post intervention	Pre-intervention	Post intervention	Pre-intervention	Post intervention
CON group (n=50)	20.33±1.23	21.85±2.33	8.36±1.22	7.85±1.43*	9.53±1.41	9.31±1.49
MED group (n=50)	20.28±1.19	25.74±2.69*	8.43±1.31	6.97±1.57*	9.42±1.39	9.04±1.69
t	0.207	-7.729	-0.277	2.930	0.383	1.610
P	0.837	<0.001	0.783	0.004	0.695	0.111

Values are reported as mean ± SD. *P<0.05 vs. Pre-intervention.

*ADL scores before and after medication reforming*

There was no significant difference in physical self-care ability and instrumental activities of daily living scores before intervention between the two groups (P > 0.05). After the intervention, the physical self-care ability of the MED group was lower than that of the CON group (P < 0.05), while the instrumental activities of daily living scores of the two groups were not significantly different after the intervention (Table 4).

*Swallowing function after medication reforming*

There was no significant difference in swallowing function before intervention between the two groups (P > 0.05). After the intervention, there was a significant difference in swallowing function between the two groups (P < 0.05), and abnormal swallowing function was less in the MED group (Table 5).

**Table 5.** Comparison of swallowing function, nutritional status, incidence rate of adverse drug reactions and satisfaction rate of family members and caregivers between the two groups.

Group	Swallowing function			Nutritional status		Incidence of adverse drug reactions	Satisfaction rates				
	Normal	Questionable	Abnormal	Well-nourished	Malnutrition		Very satisfied	Satisfied	Fair	Not satisfied	Overall satisfaction
CON group (n=50)	22 (44.0)	10 (20.0)	18 (36.0)	25 (50.0)	25 (50.0)	27 (54.0)	5 (10.0)	12 (24.0)	22 (44.4)	11 (22.2)	17 (34.0)
MED group (n=50)	33 (66.0)	10 (20.0)	7 (14.0)	35 (70.0)	15 (30.0)	11 (22.0)	18 (36.0)	28 (56.0)	3 (6.0)	1 (2.0)	46 (92.0)
x^2^			7.040		4.167	10.866					36.079
P			0.030		0.041	0.001					<0.001

Values are reported as n (%).

*Nutritional status before and after medication reforming*

There was no significant difference in nutritional status before intervention between the two groups (P > 0.05). After the intervention, the proportion of well-nourished patients was higher in the MED group than in the CON group (P < 0.05) (Table 5).

### Incidence of adverse drug reactions during treatment 

A total of 38 cases of adverse drug reactions occurred in the 100 study subjects during treatment, an overall incidence of 38.0 %. Among them, there were 7 cases of hyperkalemia, 2 cases of hypokalemia, 5 cases of hyponatremia, 3 cases of hypoglycemia, 2 cases of obstructive sleep apnea-hypopnea syndrome (OSAHS), 11 cases of hypernatremia, 1 case of sinus bradycardia, 1 case of sinus tachycardia, 1 case of gastrointestinal bleeding, 2 cases of choking, 2 cases of falls, and 1 case of fracture. There was a significant difference in the incidence of adverse drug reactions between the two groups (P < 0.05), and it was lower in the MED group (Table 5).

### Satisfaction of family members and caregivers 

There was a significant difference in satisfaction rate of family members and caregivers between the two groups (P < 0.05), and the MED group was higher than the CON group (Table 5).

## DISCUSSION

Drug reorganization can seamlessly connect the medication of patients during admission, transfer, and referral and ensure the safety, accuracy and continuity of medication. China's drug restructuring started late, lacked a good implementation process and service model, evaluation criterion and evaluation links, and had an unclear evaluation, which was still in the groping stage. Previous studies^14^^,^^15^ have demonstrated that medication reconciliation brings certain benefits to patients and medical staff and can effectively guide medication, improve treatment effect, and ensure safety. However, there is no study on drug reorganization service for hospitalized patients with AD. There is a high incidences of AD in the elderly population. Most patients have two or more underlying diseases. Medication is complex and prone to deviation, thus affecting the therapeutic effects, medication safety, and therapeutic benefits. 

In this study, we found that the types and amounts of medication in the MED group were less than those in the CON group and after compared to before the intervention. This was similar to the findings of Digiantonio et al.^16^, in which they found that the use of a pharmacy-led medication reconciliation program reduced the number of significant, serious, and life-threatening adverse effects. Scholars have reported^17^ that there is a positive correlation between the type of medication and the occurrence of medication deviation. The more types of medication involved, the more the medication deviation. Therefore, medication reconciliation for the treatment of elderly patients with AD in clinical practice can effectively reduce the types of drugs taken at the same time, and then save treatment costs and alleviate the economic pressure of patients. Medication deviation was the most important reason for medication reconciliation. In this study, the potential medication deviation was 45.0%, higher than 25.82% in the study by Nie et al.^18^, which may be related to the combination of multiple underlying diseases in elderly patients with AD as well as the side effects of taking psychotropic drugs. In this study, the incidence of medication deviation in the MED group was lower than that in the CON group. This could suggest that medication reforming in elderly patients with AD can effectively reduce medication deviation, avoid potential medication errors, ensure the accuracy, effectiveness and continuity of drug treatment and facilitate the implementation of medication reforming in elderly patients with AD in China in the future.

In terms of therapeutic effect, the cognitive ability, physical activity ability, dysphagia and nutritional status of AD patients in the MED group were improved to a certain extent after the intervention. The results suggest that effective medication reforming can reduce medication deviation to the greatest extent, improve the effectiveness of drug treatment, relieve clinical symptoms and prevent disease progression. The scientific and effective medication information can help drug reorganization and implementation in clinical practice. It is necessary to strengthen the attention to drug reorganization, improve the status and role of clinicians and clinical pharmacists in drug adjustment during hospitalization, transfer and discharge of patients to ensure effective treatment and medication safety^19^. Moreover, important measured include the establishment of an experienced drug reorganization team with the participation of medical staff and family members and of a scientific drug reorganization process and standard to scientifically evaluate the drug treatment process^20^.

Medication reforming intervention effectively reduces the combination of multiple drugs. It reduces the impact of interactions between multiple drugs while also reducing the damage of some drugs to liver and kidney functions. Therefore, effective drug reorganization is important in AD patients. In this study, the incidence of adverse drug reactions in the MED group was 22.0%, which was lower than that in the CON group (54.0%), consistent with the results of Zhao et al.^21^. These figures suggest that medication reforming can prevent the occurrence of adverse drug reactions and promote safe, standardized and long-term drug treatment of patients by reducing medication deviations and avoiding uncomfortable side effects. Further, the satisfaction rate of the MED group was higher than that of the CON group, suggesting that the family members of AD patients and the main caregivers had better satisfaction with the current drug reforming service. However, the existing medication reforming service could be further adjusted and improved, as some participants were still dissatisfied.

The innovation of this study was to explore the medication reforming service model for the first time with AD patients as the target population. This was done by thoroughly analyzing the medication problems of AD patients and proving that the incidence of medication deviation was high in these patients. The application of the Beers and STOPP/START standards to the medication reforming model effectively reduced the unintentional medication deviation and the incidence of adverse drug reactions. In addition, the participation of the patient's family members and main caregivers throughout the whole process of the drug reforming service and the effective evaluation of the advantages and disadvantages of the reforming service were also strengths of this study. However, this study also had several shortcomings. First, the sample size was small and from a single center, which could lead to selection bias. Second, the list of drug in use before reorganization was provided by family members or main caregivers, which may involve recall bias and affect the accuracy of the drug list. Third, the influencing factors of medication deviation were not explored in-depth. These need to be addressed in future multicenter studies with larger sample sizes.

In conclusion, drug reorganization can reduce medication deviation, improve therapeutic effects, prevent adverse drug reactions and ensure standardization, normalization and accuracy of medication for AD patients.
